# African Swine Fever in Wild Boar (Poland 2020): Passive and Active Surveillance Analysis and Further Perspectives

**DOI:** 10.3390/pathogens10091219

**Published:** 2021-09-19

**Authors:** Maciej Piotr Frant, Anna Gal-Cisoń, Łukasz Bocian, Anna Ziętek-Barszcz, Krzysztof Niemczuk, Grzegorz Woźniakowski, Anna Szczotka-Bochniarz

**Affiliations:** 1 Department of Swine Diseases, National Veterinary Research Institute, Partyzantów Avenue 57, 24-100 Puławy, Poland; anna.gal@piwet.pulawy.pl (A.G.-C.); grzegorz.wozniakowski@umk.pl (G.W.); anna.szczotka@piwet.pulawy.pl (A.S.-B.); 2 Department of Epidemiology and Risk Assessment, National Veterinary Research Institute, Partyzantów Avenue 57, 24-100 Puławy, Poland; lukasz.bocian@piwet.pulawy.pl (Ł.B.); anna.barszcz@piwet.pulawy.pl (A.Z.-B.); 3 National Veterinary Research Institute, Partyzantów Avenue 57, 24-100 Puławy, Poland; Krzysztof.Niemczuk@piwet.pulawy.pl; 4 Department of Diagnostics and Clinical Sciences, Faculty of Biological and Veterinary Sciences, Nicolaus Copernicus University in Toruń, Lwowska 1 Street, 87-100 Toruń, Poland

**Keywords:** ASF, wild boar, passive and active surveillance

## Abstract

African swine fever (ASF) is a fatal hemorrhagic disease of wild boar and domestic pigs which has been present in Poland since 2014. By 2020, the ASF virus (ASFV) spread across Central, Eastern and Western Europe (including Germany), and Asian countries (including China, Vietnam, and South Korea). The national ASF eradication and prevention program includes continuous passive (wild boar found dead and road-killed wild boar) and active (hunted wild boar) surveillance. The main goal of this study was to analyze the dynamic of the spread of ASF in the wild boar population across the territory of Poland in 2020. In that year in Poland, in total 6191 ASF-positive wild boar were declared. Most of them were confirmed in a group of animals found dead. The conducted statistical analysis indicates that the highest chance of obtaining an ASF-positive result in wild boar was during the winter months, from January to March, and in December 2020. Despite the biosecurity measures implemented by holdings of domestic pigs, the disease also occurred in 109 pig farms. The role of ASF surveillance in the wild boar population is crucial to apply more effective and tailored measures of disease control and eradication. The most essential measures to maintain sustainable production of domestic pigs in Poland include effective management of the wild boar population, along with strict implementation of biosecurity measures by domestic pig producers.

## 1. Introduction

African swine fever (ASF) presents a serious threat to domestic pig production worldwide. In spite of 100 years passing since the first confirmation of ASF in Africa, the disease has spread among numerous European and Asian countries [1,2,3] and recently also in the Dominican Republic [4]. An occurrence of ASF in any affected country results in serious restrictions for pig producers including export and movement limitations. There is no direct risk for human heath associated with the disease; however, there is a serious threat for pork production, which is one of the biggest industry sectors in a number of European countries, including Poland. Therefore, ASF may directly cause enormous economic losses [3,5]. Due to the serious threat for pig production and the international consequences of its introduction, ASF is a notifiable disease under strict surveillance and vigilance of the World Organization for Animal Health (OIE) and the European Union. All outbreaks (both in domestic pigs and wild boar) are subject to notification [2].

African swine fever virus (ASFV) is the only known member belonging to the *Asfarviridae* family. The virus causes a highly lethal, hemorrhagic disease which affects domestic pigs, wild boars and the African wild *Suidae* [1]. Incubation period of ASF in affected animals is usually 4–19 days (in case of acute form it takes 3–4 days). The incubation period in wild boars takes approximately 15 days. In domestic pigs, the mortality rate often approaches 100%; however, in wild boar, the mortality rate is up to 95%. [1,2,3]. ASFV is an enveloped virus with linear double-stranded DNA (dsDNA) with a length of 170 to 193 kilobase pairs (kbp) and between 150 and 170 genes (depending on the virus isolate) [6]. The genome of ASFV has all of the nucleotide sequences required for enzyme synthesis used in the process of transcription, replication and synthesis of the structural proteins that take part in virus assembly. There are also genes involved in the synthesis of factors responsible for the evasion of host defense systems [7].

Within 6 h after infection, ASFV replicates in the cytoplasm of the mononuclear-phagocytic system cells [7]. To date the genetic divergence of ASFV has resulted in 24 distinct genotypes being identified, mainly based on sequencing of the gene encoding the major capsid p72 protein. Only two genotypes have been detected outside of the African continent. Genotype I occurred in Sardinia, while genotype II is still emerging in central and Eastern Europe, as well as in Asia [8]. Despite some advances in understanding the diversity and sequencing of the whole ASFV genome in detail, there are still gaps in the knowledge concerning *i.a.* the transmission of ASFV and the direction of its evolution, as well as more specific questions related to the host-specific receptors required for the infection [9].

The wild boar (*Sus scrofa*) is the main source of ASF in Europe [10]; however, in Africa, apart from autochthonous wild *Suidae*, there is another important biological vector of ASFV represented by soft ticks belonging to the *Ornithodoros* genus [11].

In Africa, the sylvatic cycle is mainly observed in which the virus passes from a tick to a wild pig belonging to the *Suidae* family, and again to the next tick [12]. In contrast, in Europe, ASFV is mainly transmitted by direct contact between an infected host (wild boar or domestic pig) and a susceptible animal. The indirect transmission by contaminated materials such as carcasses, feed, soil, food, fomites, vehicles, and others is also possible. The second route connected with careless human activity is the most common way to introduce ASFV to domestic pig farms [13]. The high stability of the virus in the environment, resulting from its resistance to extreme pH and temperature conditions, favors its transmission [14].

The course of infection in pigs can be different and depends mainly on the ASFV isolate and biological determinants such as age and the immune system’s ability to fight infection [9]. The disease may be present in different forms, but in the Eastern Europe, the most common is the acute form with symptoms such as high fever, respiratory and gastrointestinal signs, cyanosis, numerous deaths caused by ataxia. Many animals die before any clinical signs appear because of a short virus incubation period, which in this case is 3–4 days. Due to the similarity of the indicated symptoms to other swine infectious diseases, such as classical swine fever (CSF) or porcine reproductive and respiratory syndrome (PRRS), it is very important to diagnose the diseases with a method that exhibits high sensitivity and specificity [15].

The path to the introduction of an effective vaccination seems to be long, despite numerous projects focused on this subject [16,17]. Vaccines based on inactivated viruses do not induce the production of antibodies at a satisfactory level to guarantee acquisition of resistance by the host. In turn, vaccines based on attenuated viral strains do not provide protection against all strains of the virus. Vector-based vaccines are still virulent and often cause subclinical infection, which could be transferred to another susceptible animal [15,18]. Limited knowledge of the complexity of the virus, its virulence factors and the immune mechanisms of an infected animal mean that at present, a vaccine cannot be used to safely control ASF [17]. Basic activities in the fight against ASF include careful monitoring of wild boars in the areas affected by the disease, appropriate diagnostics, and compliance with the recommended sanitary measures. Correct diagnostics is carried out with detection of the ASFV genome by PCR, detection of antibodies against viral antigens, and virus isolation [19]. Currently, there are many validated assays for ASF diagnostics, but each interpretation of the result should be carried out in accordance with accurate knowledge of the current epidemiological situation, circulating strains, its mechanisms of spread, etc. [20,21].

The situation of the ASF epidemic in the world is changing rapidly from year to year. Only in 2020 has the disease successively entered new countries such as Serbia (January), Greece (February), Papua New Guinea (March), India (May) [22] and Germany (September) [23]. Fundamental differences between regions have been observed. On the European continent, outbreaks are mostly recorded in wild boars, while in Asia, the majority of outbreaks occur in pigs, and in Africa, they are only found in pigs. Asia suffers the greatest loss of animals with 82% of the whole world’s recorded losses. This number may be due to the fact that Asian countries have the largest share in pork production [22].

Since 2007, when ASFV genotype II was discovered in Georgia, the virus has been successfully spreading across all of Eastern Europe. In Poland It is still active, despite implementation of control measures [24]. From the beginning of the ASF epidemic in Poland, which occurred more than 7 years ago, the virus has been covering new areas by spreading in the wild boar population [25]. Initially, ASF was recorded only in the eastern part of Poland, but since November of 2019, the situation has changed rapidly [23]. A study of a wild boar killed in a traffic accident carried out at the National ASF Reference Laboratory in Pulawy confirmed the first case of ASF in Lubuskie province. Searching the affected area after first outbreak, including two neighboring provinces (Wielkopolskie and Dolnośląskie), brought further confirmation of ASF cases among wild boars. The virus quickly spread to other counties around the Wschowa county where the first wild boar was found. The situation was worrying due to the proximity of these regions to the territory of Germany, which is the largest swine producer in the European Union [25].

Despite the application of measures on the Polish and German side of the border to prevent the entry of the virus into German areas, such as solid fences against wild boars, the threat of ASF was serious. On 10 September 2020, the Friedrich-Loeffler Institute confirmed the first ASF-positive result in a wild boar sample from Brandenburg state, which neighbors Poland, at a distance of about 6 km from the Polish border. By the 24th of September 2020, 32 wild boar cases were confirmed in Germany [23].

In 2020, Czechia and Belgium proved that the implementation of strict measures in the fight against ASF can bring the expected results. Belgium confirmed its last ASF wild boar outbreak in March, and almost 9 months later, the European Commission restored to Belgium its ASF-free status. It and Czechia eradicated the disease within two years. They stopped the epidemic in the wild board population mainly by implementing three important activities: building fences that limit wild boar migration, searching for the remains of dead animals (passive surveillance), and reducing the number of wild boars within the fences (active surveillance) [2].

The main concern of this work was to analyze the dynamic of the spread of ASF in the wild boar population across the territory of Poland in 2020. To determine and track the changes in the movement of the virus in the environment, field samples from the whole country were analyzed.

## 2. Results

In this study, a total of 153,057 wild boars were analyzed. The animals were divided into three separate groups: found dead (8669), road-killed (8950), and hunted (135,438). Any wild boar whose carcass was found in the environment after the natural death of the animal was included in the first group (found dead). In the second group (roadkilled) were wild boars killed in traffic accidents (killed by cars). The third group (hunted) comprised animals shot by professional hunters from the Polish Hunting Association. All of the samples were collected for the Polish ASF monitoring program (2020). In the study, a total of 6191 ASF-positive wild boars were analyzed, and 146,866 ASF-negative wild boars. All detected ASFV samples belonged to genotype II.

**Figure 1:**
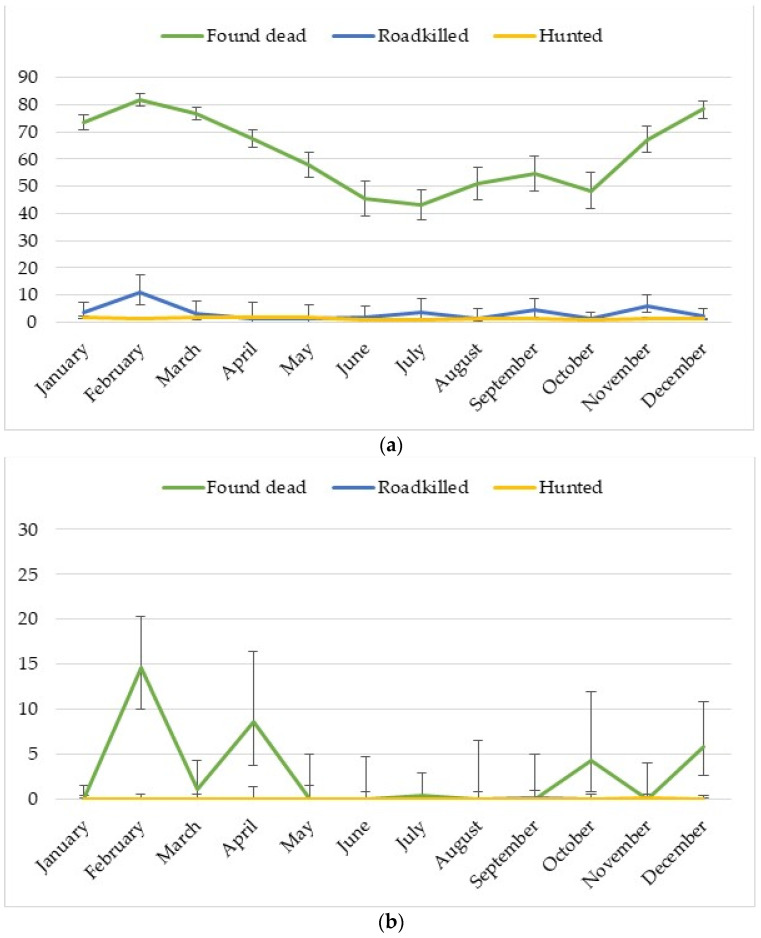
Distribution of ASF outbreaks in wild boar population: (**a**) zones II and III; (**b**) zones 0 and I.

The distribution of ASF-positive results regarding each month of 2020 and the ASF zones is presented in Figure 1. Most of the ASF-positive results are connected to the “found dead” group and with zones II and III. In Zones 0 and I, most of the positive results were also related to “found dead” animals. The month with the highest number of ASF-positive results was February. Most of the confirmed ASF wild boar outbreaks were identified in all winter months: December, January, and February, and in also cold month: March. The supplementary materials contain detailed results (Tables S1–S3).

**Figure 2:**
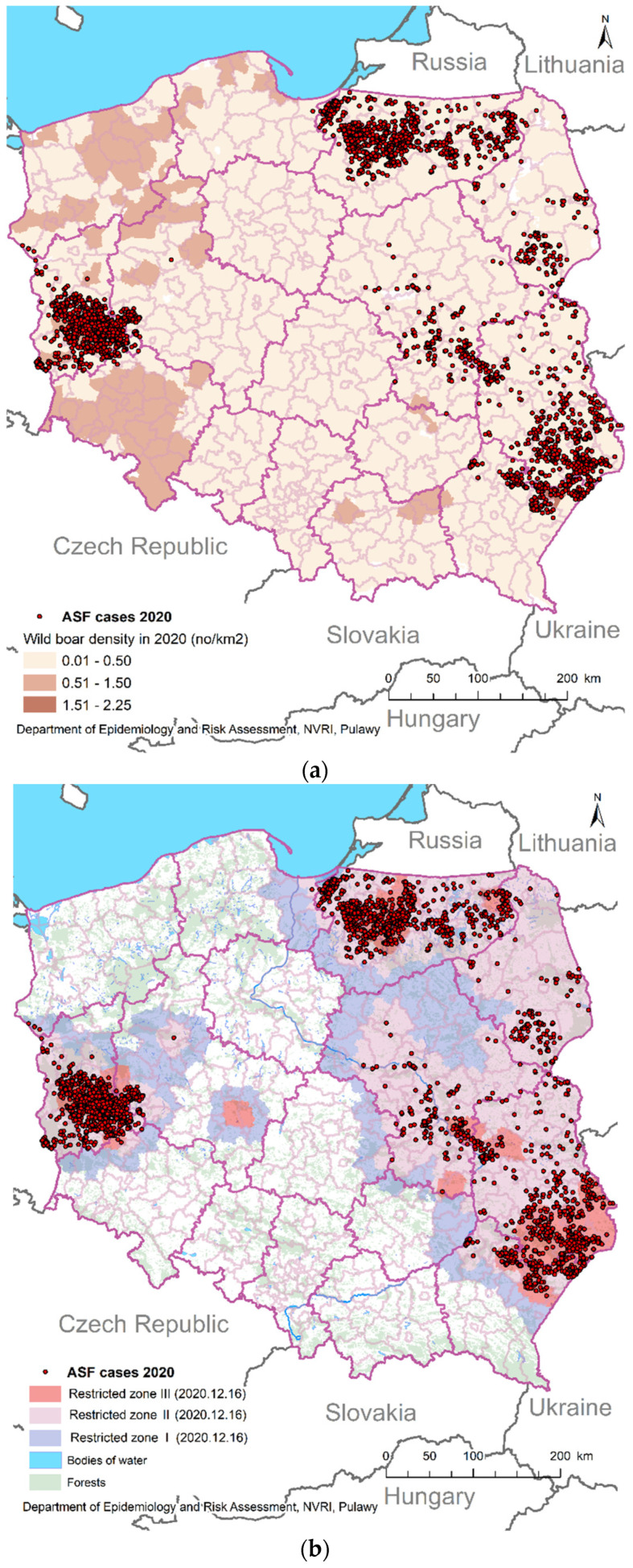
ASF outbreaks in wild boars in Poland (2020): (**a**) ASF cases compared to wild boar density; (**b**) ASF cases compared to forests (map includes ASF zones).

In 2020, a higher density of wild boar population was observed in the area where ASF was absent (highest in the Dolnoslaskie and Zachodniopomorskie provinces; Figure 2a).

Wild boars positive for ASF were concentrated in ten Polish provinces, but the highest number of them was identified in Lubuskie province (west of Poland), Warminsko-Mazurskie province (north of Poland) and Lubelskie province (east of Poland), leaving an ASF-free area in the middle of Poland (Figure 2). No connection with forestry and ASF-positive wild boars was observed (Figure 2b).

### 2.1. Passive Surveillance in 2020–ASF Outbreaks in Wild Boars (Found Dead)

In passive surveillance in the group of found-dead animals, 8669 wild boars were analyzed in total. In ASF zones II and III, 7217 animals were analyzed, of which 5008 were ASF-positive (69.4%). In ASF zones 0 and I, it was 1452 animals, of which 52 were ASF-positive (Table S1).

Logistic regression modeling showed a significant influence of the month on the prevalence of ASF (*p* < 0.0001) in zones II and III. Most of the months were significantly different from the reference month of July, when the prevalence was the lowest (43.1%). The chances of obtaining a positive result in the wild boar population were the highest in February (6 times), December (5 times), March (4 times), and January (3.5 times) compared to reference the month (Table 1).

As in the case of zones II and III, the model showed a significant influence of the month on the prevalence level (*p* < 0.0001) in zones 0 and I. Due to the lack of positive results in January, May, June, August, September and November, these months were not included in the model. Of the remaining months, July was selected as the reference month with the lowest prevalence (0.5%). In February, April and December, the percentage of positive results and the chance of obtaining a positive result were significantly higher than in July (Table 1; Table S1; Figure 1b).

### 2.2. Passive Surveillance—ASF Outbreaks in Wild Boars (Roadkilled)

In passive surveillance in the group of roadkilled animals, a total of 8950 wild boars were analyzed. In ASF zones II and III, 1888 animals were analyzed, of which 68 were ASF-positive (3.6%). In ASF zones 0 and I, it was 7062 animals, of which only one was ASF-positive (Table S2).

The models indicate a significant influence of the month on the level of prevalence (*p* = 0.0001) in ASF zones II and III. However, the percentage of positive results (17.4%; Table S2, Figure 1a) was only significantly higher in February than in the reference month May, when the prevalence was the lowest. The chance of obtain an ASF-positive result in February was more than 10 times higher than in the reference month (Table 2).

In the case of zones 0 and I (roadkilled), it was not possible to create a model for the data, as the only positive result was detected in September (prevalence 0.2%; Figure 1b, Table S2).

### 2.3. Active Surveillance—ASF Outbreaks in Wild Boars (Hunted)

In active surveillance (hunted animals), a total of 135,438 wild boars were analyzed. In ASF zones II and III, 83,373 animals were analyzed, of which 1053 were ASF-positive (1.2%). In ASF zones 0 and I, it was 51,012 animals, of which 9 were ASF-positive (Table S3).

The model for ASF zones II and III showed a significant influence of the month on the level of prevalence (*p* < 0.0001). In all months except for June and July, the percentage of positive results was significantly higher than in the reference month of October, where the prevalence was 0.8% (Table S3, Figure 1a). The chances of obtaining an ASF-positive results in January, February, March, April, May, November and December were 2 times higher than in the reference month (Table 3).

A model was built for March, November, December and the reference month of July for ASF zones 0 and I after excluding the non-ASF-positive months. However, only a low level of significance (*p* = 0.18) was obtained, which proves that the influence of the month on the level of the percentage of positive results is not significant in the analyzed group (Table 3).

### 2.4. ASF-Positive Results Regarding Animal Status (Found Dead, Roadkilled, Hunted)

The model indicates importance of the status of the wild boar population regarding the level of prevalence of ASF (*p* < 0.0001) in all ASF zones. In zones II and III, the chance of obtaining a positive result in a wild boar found dead was 166 times higher than among the hunted animals. In case of roadkilled wild boars, the chance was 3 times higher than in the reference group (Table 4). Similarly, in zones 0 and I, for the found dead animals, the chance of obtaining a positive result was 210 times higher than in the hunted group. In the group of roadkilled animals, no differences were observed (Table 4).

### 2.5. Comprehensive Model for ASF-Positive Wild Boars

The comprehensive model showed a significant impact of the month, ASF zone, and animal status on the prevalence level (*p* < 0.0001). All months except for July (*p* = 0.25) were significantly different from the reference October, in which the prevalence was the lowest (Table 5). In general, the chances of obtaining positive results were highest in the winter months: January (3 times), February (4 times), March (3 times), and December (3 times) than in reference month of October (Table 5).

In the case of ASF zones, the chance for a positive result in the wild boar population was significantly higher in ASF zones II and III than in zones 0 and 1 (Table 5).

Most of the ASF-positive cases in the wild boar population were identified in the group animals found dead (Figure 1, Table S1). Similarly, the chance of obtaining an ASF-positive result was the highest in the group of wild boars found dead (150 times higher than in the hunted group, *p* < 0.0001, Table 5).

## 3. Discussion

Poland has been struggling with ASF since February 2014 [10]. From that moment, a successively increasing number of ASF outbreaks in the wild boar population has been observed [10,24,26]. In 2017, 1121 ASF-positive wild boar samples were found, and in 2018, it was 3936 samples [25]. The year 2019 brought 3830 ASF-positive animals [24], while in 2018–2019, there was a stabilization in the number of ASF outbreaks in wild boars [24,26]. However, in 2020, the situation dramatically changed with an increased number of ASF-positive wild boars (*n* = 6,191). (Tables S1–S3). In the same period, a similar increase was observed in Hungary: 1430 ASF outbreaks in wild boars in 2019, and 3422 in 2020 [3].

In 2019 in Poland, a long-distance ASF introduction to the Lubuskie province was observed, hundreds of kilometers away from previous outbreaks [24,25]. Last year, no such long-distance ASF jump like the one in 2019 was noted. The disease spread a short distance from previous outbreaks, and this spread was limited to the areas neighboring zones II and III (Figure 2). The increased number of ASF outbreaks in wild boars in Lubuskie province (Figure 2) affected the neighboring country: Germany. On 10th September 2020 in Germany, the first ASF outbreak in wild boars was confirmed. The carcass was located in Brandenburg, approx. 6 km from the Polish border [23]. It seems that the slow virus spread across the Baltic States and Poland is related to the fact that the average distance of wild boar migration is from 8 to 17 km per year [11]. Schulz et al. (2019) also indicated that even the highly virulent strains of ASFV (circulating in Eastern and Central Europe) might be characterized by low morbidity, affecting the slow spread of ASF in the wild boar population [27].

Most ASF-positive results were found in the group of dead animals. A similar situation is observed in other European countries, including Hungary and Romania, where the total number of ASF wild boar outbreaks were connected with passive surveillance [3,11]. In that group of animals, the highest chance of obtaining a positive result was observed in the colder months (February, December, March, and January) in Poland (Table 1, Figure 1). Similar phenomena were observed by other authors in Belgium and Slovakia (January, February, March), Czechia and Estonia (October, November, December), Hungary (February, March April), and Lithuania and Latvia (November, December) [3]. A team of Lithuanian scientists analyzed data from 2014 to 2018 and showed an increase of ASF outbreaks in wild boars in the winter season (December–February) in every analyzed year [28].

In the European Union and in neighboring countries affected by ASF, a program of active and passive surveillance was implemented [3,11,24]. The chance of obtaining a positive result in the wild boar population in Poland was 150 times higher in 2020 in the group of animals found dead than in the hunted group (Table 5), which indicates the advantage of passive overactive surveillance. Analogical results were presented in previous years by Pejsak (2018) [10], and Frant (2020, 2021) [24,26]. The deterministic SIR model developed by Gervasi et Al. (2019) also indicates the main role of passive surveillance in the early detection of ASF [29]. The predominance of activities related to collecting carcass from the forest environment was also demonstrated in Lithuania [28,30], Latvia [31], and Estonia [32]. Although the total number of analyzed hunted wild boars from Latvia was higher than from the group of dead animals, the chance of obtaining a positive result was definitely greater in group of collected carcasses [31], which resembles the situation in Poland.

Another part of the monitoring program of passive surveillance in Poland is the group of road-killed animals. In our comprehensive model, we observed an almost 3 times higher chance of obtaining a positive result than in the hunted group. The researchers from Estonia and Latvia have tried to compare these two groups in their country; however, the total number of samples in the roadkilled group was too low to draw any statistical conclusions [33].

In the case of active surveillance, in which the total number of analyzed samples was the highest, a significance differences in seasonality of ASF in the wild boar population was not observed. A situation similar to the Polish one was observed in other European ASF-affected countries. The prevalence of hunted animals from our country was 1.2%, which is a similar result to those obtained in Belgium (0.9%), Hungary (1.3%), and Romania (1.2%) in the whole epidemic period [3].

The Romanian data from the analysis of *Stomatoxys* and *Culicoides* collected from domestic pig outbreaks indicate a potential role of insects in spreading ASF [34]. However, the lack of a significant increase in ASF-positive wild boars in the summer season seems to exclude the role of insects in spreading the disease, at least in Poland. The wild boar population density is a another factor which has a significant influence on the spread of ASF among wild boars [35]; however, in 2020 (Figure 2a) and in 2019 in Poland, the highest density of wild boars were seen in the ASF-free (zones 0 and I) areas, which could be connected with intense hunting in the ASF zones and the activity of ASFV [24].

In the winter, an increase in ASF outbreaks in the Polish wild boar population was observed. This could be connected with the longer survival time of the virus in the carcasses. Presumably, another reason may be thick forests vegetation covering the carcasses in the summer [3]. In addition, in the winter, the food sources and water for wild boars are limited, which could affect wild boars’ condition [11]. Aggressive interactions between wild boars during the mating season from October to January may also be of consequence for the spread of the disease during the colder months [36].

The ongoing spread of ASFV in the wild boar population in Poland is still taking place. Unfortunately, last year (2020), the total number of ASF outbreaks in wild boars was almost twice as high as in the previous year (2019). Action is being taken by the Polish government and the Veterinarian Inspectorate to slow the spread of the virus in Poland; however, the disease is constantly dangerous and active. Passive and active surveillance makes it easier to track new wild boar ASF outbreaks, which immediately translates into virus prevention in pig herds by reducing the virus pressure in the environment. The above-mentioned activities, along with appropriate biosecurity measures, enhance the protection of herds. The role of monitoring for ASF in the wild boar population is very important, and the actions performed toward virus reduction are essential for the safety of pork production in Poland.

## 4. Materials and Methods

All laboratory studies (sample preparation, DNA extraction, molecular and serological analyses) were conducted in a biosafety level 3 laboratory (BSL-3), in a reference laboratory for ASF in Poland by qualified technicians and researchers.

The analyzed material were field (environmental) samples from wild boars from Poland (ASF zones 0, I, II, III). ASF zone 0 is the area where the ASF is not present and there are not implemented additional restrictions. ASF zones I, II and III are responding zones to the European Union legislation: restricted zones I (protection area), II (ASF confirmed in wild boars) and III (ASF confirmed in domestic pigs) [37,38]. For the tests, blood/serum, bone marrow, and tissue samples (e.g., tonsil, spleen, kidney, lung) were used. All types of samples were taken for found dead, roadkilled and hunted group, however for the passive surveillance (found dead and roadkilled) most of them were bone marrow and tissue material and for the active surveillance (hunted) most of them were blood/serum samples. The material was collected by the local veterinary inspectorate employees within the ASFV monitoring programme in Poland and were analyzed for the presence of ASFV DNA and/or antibodies against ASFV using virological (molecular), and serological methods.

For molecular detection, real-time PCR was used, as described below. Before the analysis, sample preparation was conducted. Tissue sections were homogenized in phosphate-buffered saline (PBS) yielding a 10% homogenate. Next, DNA extraction with the use a QIAamp DNA Mini Kit (QIAGEN, Germany), or a QIAcube HT system (Indical, Germany) was performed. The positive control of DNA extraction was provided by the European Union Reference Laboratory (EURL) for ASF (CISA-INIA, Valdeolmos, Spain).

Real-time PCR was conducted by the method described by Fernandez-Pinero [19] or with the use of one of the following commercial kits: Virotype (Indical, Germany), ID Gene African Swine Fever Duplex (IDvet, France). The amplification process was conducted in one of four types of thermocyclers (Applied Biosystems 7500, Applied Biosystems, USA; QuantStudio™ 5, ThermoFisher Scientific, USA; Rotor-Gene Q, QIAGEN, Germany; LightCycler 480, Roche, Switzerland). A fluorescent signal with a threshold cycle value (Ct) below 37.0 was considered as positive.

In order to obtain serum from the blood, before the analyses, the whole blood was centrifuged in 8961514× *g*. The serological status of the serum samples was determined by the enzyme-linked immunosorbent assay (ELISA). For that method, a commercial kit: ID Screen^®^ African Swine Fever Indirect (IDVet Innovative diagnostic, France) as used. The analyses were conducted according to the manufacturer’s procedure.

Every positive and doubtful result obtained in the ELISA test was verified with a confirmation test: the indirect immuno-peroxidase technique (IPT). Test procedure is more sensitive (approximately 100 times) than ELISA. The result of the method is observed in a reverse-field microscope. The IPT reagents were provided by the EURL for the ASF (CISA-INIA, Valdeolmos, Spain) and the analysis was conducted according to the EURL protocol.

For the surveillance calculation, each positive result obtained in qPCR (ASFV DNA) and/or IPT (anti-ASFV antibody) was consider positive.

Examination of the ASF frequency in wild boars during 2020 was estimated separately for every month and individually in each category:

- passive surveillance (found dead), zones II–III;
- passive surveillance (road-killed), zones II–III;
- active surveillance (hunted), zones II–III;
- passive surveillance (found dead), zones I–0;
- active surveillance (hunted), zones I–0;
- passive surveillance (road-killed), zones I–0.

Moreover, examination of the prevalence of ASF was conducted separately for wild boars found in different conditions (found dead, road-killed, hunted) in the following categories:

- zones II–III;
- zones 0–I.

The ASF zones (0–I–II–III) were designed according to 2014/709/EU decision [37] (since 21 April 2021 for ASF zones a new legislation is in the force 2021/605/EU) [38].

In addition, a comprehensive model for the year 2020 was constructed, which analyzed all factors taken simultaneously—months, wild boar status, and the zone from which the sample was collected.

The statistical analyses were conducted with the application of logistic regression models. This type of model is a mathematical formula that can be used to report the effect of several variables (*X*_1_, *X*_2_, …, *X*_n_) on the dichotomous variable *Y*, which has one of two possible rates (in this case: positive or negative):

(1) $$P\left(Y=1|x_{1},x_{2},\ldots ,\;x_{n}\right)=\frac{e^{\left(\beta_{0}+\sum_{i=1}^{n}\beta_{i}x_{i}\right)}}{1+e^{\left(\beta_{0}+\sum_{i=1}^{n}\beta_{i}x_{i}\right)}}$$

where:

*β_i_*—regression coefficient for *i* = 0, …, *n*,
*x_i_*—independent variables (measurable or qualitative) for *i* = 1,2, … *n*.

In order to obtain the ratings of the coefficients, the maximum likelihood method was used. The significance of the independent variables was estimated using the Wald test. The fit of the model to the data was also determined in advance using the likelihood ratio (LR statistics). Odds ratios (ORs) were established with 95% confidence intervals.

For the logistic regression model, the described relationships were statistically demonstrated at the adopted significance level of α = 0.05.

All statistical analyses were conducted with the use of TIBCO Software Inc. (2017) Statistica (data analysis software system), version 13. The monthly distributions of ASF-positive wild boars (Figure 1) were made in Excel 2016 (Microsoft, USA). The geographical distribution of ASF wild boar outbreaks (Figure 2) were made in ArcGIS 10.4.1 (ESRI).

## Figures and Tables

**Table 1:** Results of logistic regression models. Passive surveillance model for dead wild boars found.

Significance Assessment of the Model (*p* Value of the LR ^1^ Test)	Independent Variable	Coefficient (βi)	Std. ^2^ Error	*p* Value (Wald)	Odds Ratio	Confidence OR ^3^ − 95%	Confidence OR ^3^ + 95%
Wild boars found dead in ASF zones II and III—Impact of the month on the result (reference month: July)							
<0.0001	Absolute term (β0)	−0.27721	0.10927	0.01120	0.75789	0.61176	0.93894
	January	1.30962	0.13134	*p* < 0.0001	3.70479	2.86381	4.79273
	February	1.78336	0.13285	0	5.94983	4.58570	7.71974
	March	1.47939	0.12575	0	4.39029	3.43116	5.61753
	April	1.02127	0.13202	*p* < 0.0001	2.77674	2.14358	3.59692
	May	0.60145	0.14525	*p* < 0.0001	1.82476	1.37261	2.42586
	June	0.09489	0.16553	0.56650	1.09953	0.79485	1.52101
	August	0.31255	0.16145	0.05292	1.36690	0.99607	1.87581
	September	0.47028	0.16984	0.00564	1.60043	1.14721	2.23270
	October	0.21441	0.17187	0.21224	1.23913	0.88471	1.73553
	November	0.99842	0.15511	*p* < 0.0001	2.71398	2.00240	3.67842
	December	1.56634	0.14611	*p* < 0.0001	4.78909	3.59634	6.37742
Wild boars found dead in ASF zones 0 and I—Impact of the month on the result (reference month: July)							
<0.0001	Absolute term (β0)	−5.21495	1.00336	*p* < 0.0001	0.00544	0.00076	0.03894
	February	3.44643	1.02310	0.00079	31.38823	4.21377	233.8099
	March	0.81433	1.23021	0.50819	2.25767	0.20185	25.25229
	April	2.86356	1.06922	0.00754	17.52381	2.14891	142.9023
	October	2.10886	1.16381	0.07033	8.23881	0.83913	80.89124
	December	2.44932	1.06041	0.02114	11.58042	1.44487	92.81559

^1^ LR—logistic regression; ^2^ Std.—standard; ^3^ OR—odds ratio

**Table 2:** Results of logistic regression models. Passive surveillance model for roadkilled wild boars.

Significance Assessment of the Model (*p* Value of the LR ^1^ Test)	Independent Variable	Coefficient (βi)	Std. ^2^ Error	*p* Value (Wald)	Odds Ratio	Confidence OR ^3^ − 95%	Confidence OR ^3^ + 95%
Road-killed wild boars in ASF zones II and III—Impact of the month on the result (reference month: May)							
0.0001	Absolute term (β0)	−4.41884	0.93870	*p* < 0.0001	0.01205	0.00191	0.07594
	January	1.15527	1.01450	0.25496	3.17486	0.43411	23.21920
	February	2.32287	0.97762	0.01760	10.20492	1.50005	69.42461
	March	1.07245	1.04305	0.30400	2.92253	0.37785	22.60454
	April	0.12838	1.36178	0.92490	1.13699	0.07867	16.43156
	June	0.34981	1.18298	0.76749	1.41880	0.13941	14.43931
	July	1.12300	1.06773	0.29305	3.07408	0.37866	24.95604
	August	0.18473	1.18720	0.87636	1.20290	0.11722	12.34379
	September	1.38629	1.00593	0.16834	4.00000	0.55621	28.76604
	October	0.11478	1.05369	0.91327	1.12162	0.14202	8.85818
	November	1.69317	0.97571	0.08285	5.43668	0.80215	36.84768
	December	−0.77412	1.37270	0.57287	0.46111	0.03123	6.80812

^1^ LR—logistic regression; ^2^ Std.—standard; ^3^ OR—odds ratio

**Table 3:** Results of logistic regression models. Active surveillance model for hunted wild boars.

Significance Assessment of the Model (*p* Value of the LR ^1^ test)	Independent Variable	Coefficient (βi)	Std. ^2^ Error	*p* Value (Wald)	Odds Ratio	Confidence OR ^3^ − 95%	Confidence OR ^3^ + 95%
Hunted wild boars in ASF zones II and III—Impact of the month on the result (reference month: October)							
<0.0001	Absolute term (β0)	−4.84559	0.11826	0	0.00786	0.00623	0.00992
	January	0.84917	0.14694	*p* < 0.0001	2.33771	1.75210	3.11905
	February	0.60758	0.16236	0.00018	1.83598	1.33504	2.52489
	March	0.69791	0.14546	*p* < 0.0001	2.00955	1.51055	2.67339
	April	0.76113	0.14893	*p* < 0.0001	2.14070	1.59819	2.8673
	May	0.71365	0.14616	*p* < 0.0001	2.04141	1.53237	2.71955
	June	0.14960	0.15010	0.31893	1.16137	0.86506	1.55918
	July	0.19344	0.21676	0.37216	1.21342	0.79302	1.85669
	August	0.48992	0.16726	0.00340	1.63218	0.17547	2.26632
	September	0.40117	0.16543	0.01531	1.49357	1.07955	2.06637
	November	0.57003	0.14452	*p* < 0.0001	1.76832	1.33165	2.34818
	December	0.56180	0.13999	*p* < 0.0001	1.75383	1.33254	2.3083
Hunted wild boars in ASF zones 0 and I—Impact of the month on the result (reference month: July)							
0.17981	Absolute term (β0)	−8.68491	1.04092	*p* < 0.0001	0.00017	0.00002	0.00130
	March	0.24784	1.51409	0.86998	1.28126	0.06588	24.91773
	November	1.80844	1.13267	0.11036	6.10092	0.66254	56.17939
	December	0.65922	1.26177	0.60136	1.93329	0.16301	22.92851

^1^ LR—logistic regression; ^2^ Std.—standard; ^3^ OR—odds ratio

**Table 4:** Results logistic regression models–wild boar status.

Significance Assessment of the Model (*p* Value of the LR ^1^ Test)	Independent Variable	Coefficient (βi)	Std. ^2^ Error	*p* Value (Wald)	Odds Ratio	Confidence OR ^3^ − 95%	Confidence OR ^3^ + 95%
ASF-affected wild boars in zones II and III—Impact of animal status (reference status: hunted)							
<0.0001	Absolute term (β0)	−4.29218	0.02921	0	0.01368	0.01291	0.01448
	Found dead	5.11187	0.03880	0	165.9804	153.8082	179.1158
	Road-killed	1.06590	0.12534	*p* < 0.0001	2.90344	2.27017	3.71338
ASF-affected wild boars in zones 0 and I–Impact of animal status (reference status: hunted)							
<0.0001	Absolute term (β0)	−8.64273	0.33272	0	0.00017	0.00009	0.00033
	Found dead	5.34975	0.36133	0	210.5549	103.6786	427.6036
	Road-killed	−0.21734	1.03383	0.83349	0.80466	0.10600	6.10848

^1^ LR–logistic regression; ^2^ Std.–standard; ^3^ OR–odds ratio

**Table 5:** Results of logistic regression models—comprehensive model.

Significance Assessment of the Model (*p* Value of LR ^1^ Test)	Independent Variable	Coefficient (βi)	Std. ^2^ Error	*p* Value (Wald)	Odds Ratio	Confidence OR ^3^ − 95%	Confidence OR ^3^ + 95%
< 0.0001	Absolute term (β0)	−8.51029	0.14976	0	0.00020	0.00015	0.00027
	January	1.14926	0.09569	0	3.15587	2.61416	3.80982
	February	1.46140	0.09440	0	4.31199	3.58090	5.19233
	March	1.19758	0.09099	0	3.31211	2.76910	3.96162
	April	0.94353	0.09668	*p* < 0.0001	2.56903	2.12390	3.10745
	May	0.72345	0.10578	*p* < 0.0001	2.06153	1.67410	2.53864
	June	0.20609	0.09764	0.03480	1.22886	1.01403	1.48920
	July	0.12661	0.10891	0.25405	1.13497	0.91600	1.40628
	August	0.26906	0.11831	0.02296	1.30873	1.03688	1.65186
	September	0.38919	0.11842	0.00010	1.47579	1.16899	1.86311
	November	0.83992	0.10326	*p* < 0.0001	2.31612	1.89024	2.8381
	December	1.06682	0.09689	*p* < 0.0001	2.90612	2.40161	3.51662
	Zone 0	−4.38110	1.01662	*p* < 0.0001	0.01251	0.00169	0.09252
	Zone II	3.14131	0.13346	0	23.13404	17.79016	30.08314
	Zone III	4.08250	0.13665	0	59.29332	45.31107	77.59026
	Found dead	5.01313	0.03998	0	150.3741	138.9945	162.6853
	Road-killed	1.02114	0.13037	*p* < 0.0001	2.77635	2.14804	3.58846

^1^ LR—logistic regression; ^2^ Std.—standard; ^3^ OR—odds ratio

## Supplementary Materials

The following are available online at https://www.mdpi.com/article/10.3390/pathogens10091219/s1, Table S1: Passive surveillance (wild boar found dead), Table S2: Passive surveillance (roadkilled), Table S3: Active surveillance (hunted wild boar).
