# Supplementary material for: African Swine Fever in Wild Boar (Poland 2020): Passive and Active Surveillance Analysis and Further Perspectives
Source: Pathogens. 2021 Sep 19;10(9):1219. doi: 10.3390/pathogens10091219 (PMC8465799; doi:10.3390/pathogens10091219)
Supplement: Supplementary file 1 [file pathogens-10-01219-s001.zip › pathogens-1396541-supplementary.pdf]

Table S1. Passive surveillance (wild boar found dead).

	Parts II and III						Parts 0 and I					
Year/ month	Neg.[1]	Pos.[2]	Tot.[3]	Prev.[4] [%]	-95% Cl[5]	+95% Cl[5]	Neg.[1]	Pos.[2]	Tot.[3]	Prev.[4] [%]	-95% Cl[5]	+95% Cl[5]
**2020**	**2209**	**5008**	**7217**	**69.4**	**68.3**	**70.5**	**1400**	**52**	**1452**	**3.6**	**2.7**	**4.7**
**January**	255	716	971	73.7	70.8	76.5	230	0	230	0.0	0.0	1.6
**February**	214	965	1179	81.8	79.5	84.0	170	29	199	14.6	10.0	20.3
**March**	336	1118	1454	76.9	74.6	79.0	163	2	165	1.2	0.1	4.3
**April**	268	564	832	67.8	64.5	71.0	84	8	92	8.7	3.8	16.4
**May**	188	260	448	58.0	53.3	62.7	70	0	70	0.0	0.0	5.1
**June**	138	115	253	45.5	39.2	51.8	77	0	77	0.0	0.0	4.7
**July**	190	144	334	43.1	37.7	48.6	184	1	185	0.5	0.0	3.0
**August**	139	144	283	50.9	44.9	56.8	54	0	54	0.0	0.0	6.6
**September**	108	131	239	54.8	48.3	61.2	70	0	70	0.0	0.0	5.1
**October**	115	108	223	48.4	41.7	55.2	67	3	70	4.3	0.9	12.0
**November**	123	253	376	67.3	62.3	72.0	88	0	88	0.0	0.0	4.1
**December**	135	490	625	78.4	75	81.6	143	9	152	5.9	2.7	10.9

[1]Neg. – Negative; [2]Pos. – Positive; [3]Tot. – Total; [4]Prev. – Prevalence; [5]Cl – confidence interval.

Table S2. Passive surveillance (roadkilled).

	Parts II and III						Parts 0 and I					
Year/ month	Neg.[1]	Pos.[2]	Tot.[3]	Prev.[4] [%]	-95% Cl[5]	+95% Cl[5]	Neg.[1]	Pos.[2]	Tot.[3]	Prev.[4] [%]	-95% Cl[5]	+95% Cl[5]
**2020**	**1820**	**68**	**1888**	**3.6**	**2.8**	**4.5**	**7061**	**1**	**7062**	**0.0**	**0.0**	**0.1**
**January**	183	7	190	3.7	1.5	7.4	898	0	898	0.0	0.0	0.4
**February**	122	15	137	10.9	6.3	17.4	607	0	607	0.0	0.0	0.6
**March**	142	5	147	3.4	1.1	7.8	610	0	610	0.0	0.0	0.6
**April**	73	1	74	1.4	0.0	7.3	257	0	257	0.0	0.0	1.4
**May**	83	1	84	1.2	0.0	6.5	241	0	241	0.0	0.0	1.5
**June**	117	2	119	1.7	0.2	5.9	408	0	408	0.0	0.0	0.9
**July**	108	4	112	3.6	1.0	8.9	977	0	977	0.0	0.0	0.4
**August**	138	2	140	1.4	0.2	5.1	486	0	486	0.0	0.0	0.8
**September**	166	8	174	4.6	2.0	8.9	582	1	583	0.2	0.0	1.0
**October**	222	3	225	1.3	0.3	3.8	627	0	627	0.0	0.0	0.6
**November**	229	15	244	6.1	3.5	9.9	604	0	604	0.0	0.0	0.6
**December**	237	5	242	2.1	0.7	4.8	764	0	764	0.0	0.0	0.5

[1]Neg. – Negative; [2]Pos. – Positive; [3]Tot. – Total; [4]Prev. – Prevalence; [5]Cl – confidence interval.

Table S3. Active surveillance (hunted wild boar).

	Parts II and III						Parts 0 and I					
Year/ month	Neg.[1]	Pos.[2]	Tot.[3]	Prev.[4] [%]	-95% Cl[5]	+95% Cl[5]	Neg.[1]	Pos.[2]	Tot.[3]	Prev.[4] [%]	-95% Cl[5]	+95% Cl[5]
**2020**	**83373**	**1053**	**84426**	**1.2**	**1.2**	**1.3**	**51003**	**9**	**51012**	**0.0**	**0.0**	**0.0**
**January**	6800	125	6925	1.8	1.5	2.1	4703	0	4703	0.0	0.0	0.1
**February**	5957	86	6043	1.4	1.1	1.8	3119	0	3119	0.0	0.0	0.1
**March**	9050	143	9193	1.6	1.3	1.8	4615	1	4616	0.0	0.0	0.1
**April**	7723	130	7853	1.7	1.4	2.0	4045	0	4045	0.0	0.0	0.1
**May**	8099	130	8229	1.6	1.3	1.9	4186	0	4186	0.0	0.0	0.1
**June**	7337	67	7404	0.9	0.7	1.1	3994	0	3994	0.0	0.0	0.1
**July**	4926	47	4973	0.9	0.7	1.3	5913	1	5914	0.0	0.0	0.1
**August**	4987	64	5051	1.3	1.0	1.6	2905	0	2905	0.0	0.0	0.1
**September**	6301	74	6375	1.2	0.9	1.5	3066	0	3066	0.0	0.0	0.1
**October**	7122	56	7178	0.8	0.6	1.0	3491	0	3491	0.0	0.0	0.1
**November**	9134	127	9261	1.4	1.1	1.6	4846	5	4851	0.1	0.0	0.2
**December**	9355	129	9484	1.3	1.3	1.4	6120	2	6122	0.0	0.0	0.1

[1]Neg. – Negative; [2]Pos. – Positive; [3]Tot. – Total; [4]Prev. – Prevalence; [5]Cl – confidence interval.
